# Wellbeing of Family Carers of Adults With Intellectual Disabilities During the COVID‐19 Pandemic in the UK: Longitudinal Study

**DOI:** 10.1111/jir.13206

**Published:** 2024-12-24

**Authors:** Paul A. Thompson, Eleanor Summers, Sue Caton, Nikita Hayden, Stuart Todd, Edward Oloidi, Laurence Taggart, Rosemary Kelly, Jill Bradshaw, Roseann Maguire, Andrew Jahoda, Chris Hatton, Richard P. Hastings, The Coronavirus and People with Learning Disabilities Study Team

**Affiliations:** ^1^ Centre for Intellectual and Developmental Disabilities University of Warwick Coventry UK; ^2^ Department of Social Policy and Society University of Birmingham UK; ^3^ Manchester Metropolitan University Manchester UK; ^4^ University of Sheffield Sheffield UK; ^5^ University of South Wales Newport UK; ^6^ Ulster University Coleraine UK; ^7^ Institute of Nursing and Health Research University of Ulster Jordanstown UK; ^8^ University of Kent Canterbury UK; ^9^ University of Glasgow Glasgow UK

**Keywords:** COVID‐19, family‐carer wellbeing, impact of caring, intellectual disability

## Abstract

**Background:**

Longitudinal studies of family carers of people with intellectual disabilities during the COVID‐19 pandemic have been very rare. This study investigated trajectories of family‐carer wellbeing and the impact of the caring role on carers' health over four time points measured during the COVID‐19 pandemic and after all public health restrictions had been lifted (between December 2020 and late 2022) across the United Kingdom.

**Methods:**

Family carers of adults with intellectual disabilities participated through a co‐designed, online survey at four time points across the pandemic (2020–2022). Growth models were used to determine the change in family‐carer wellbeing (*n* = 312) and the impact of the caring role on carers' health across the pandemic and what factors were associated with these outcomes. We explored associations between profound and multiple intellectual disabilities (PMID), the cared‐for person's individual wellbeing, the cared‐for person's age, whether the cared‐for person lived with their family and family‐carer wellbeing and impact of caring trajectories.

**Results:**

Overall, family‐carer wellbeing improved, and the impact of the caring role on carers' health reduced across the time period. If the cared‐for person had PMID was associated with greater degrees of depression and stress for caregivers and thus increased the impact of the caring role on carers' health, but it was not associated with carer wellbeing. Similarly, the reduction in individual wellbeing of the cared‐for person and the caregiver's perception of this person's wellbeing was also significantly associated with increased impact of the caring role on carers' health and carer wellbeing. There was no evidence that age of cared‐for person was predictive of either outcome, and there were mixed findings on whether living at home was an associated factor for either outcome.

**Conclusions:**

Overall, family‐carer wellbeing improved, and the impact of the caring role on carers' health reduced across the time period, but the cared‐for persons' poorer wellbeing and complex needs (indexed by the presence of PMID) were associated with negative impacts on family carers during the pandemic period.

## Introduction

There has been a considerable research effort to examine the impact and experiences of the COVID‐19 pandemic for people with intellectual disabilities and their families. Family carers have been the population of interest in multiple research studies although scoping reviews (Doody and Keenan 2021; Keenan and Doody 2023) suggest that the vast majority of COVID‐19 research in the field of intellectual disabilities has adopted qualitative methods and cross‐sectional surveys. Longitudinal studies focused on family carers of people with intellectual disabilities in relation to the COVID‐19 pandemic have been rare although longitudinal designs, especially using quantitative methods, are crucial to address questions about the putative impact of the COVID‐19 pandemic on family‐carer outcomes and patterns of change in family‐carer outcomes throughout the course of the pandemic.

Outside the field of intellectual disabilities, in research focused on carers in general, there have been several longitudinal studies. A recent systematic review of quantitative studies of associations between unpaid caring (i.e., those who have caring responsibilities but are not formally employed or paid to do so, such as a parent, sibling or other relative) and mental health during the COVID‐19 pandemic concluded that being a carer during the pandemic was associated with a small increased risk of mental health problems (Ervin et al. 2024) compared with not having a caring role. This review included five longitudinal studies using data about mental health and about caring at one or more points before the pandemic and at least one point during the pandemic period (i.e., from March/April 2020 onwards). These five studies were ongoing cohort studies of adults or older adults.

Whitley, Beauchamp and Brown (2021) found that adults caring for someone at home during the first phase of the pandemic in the United Kingdom (April and July 2020) had higher levels of psychological distress compared with non‐carers and a greater decline in mental health. Similarly, Park (2021) found that adults in the United States who were carers reported higher levels of psychological distress early in the pandemic (April/May 2020) compared with non‐carers after controlling for immediately pre‐pandemic (January 2020) levels of psychological distress. Costi et al. (2023) also found an association between caring and increased psychological distress but only for adults who became carers during the pandemic in the United Kingdom (i.e., not for adults who continued in their pre‐pandemic caring role). In addition to the nuances in findings relating to whether care was provided to someone at home or the carer was new to their role, the two other pre‐post pandemic longitudinal studies provided contrasting findings. For older adults (60+ years), McGarrigle et al. (2022) found no association between caring during the pandemic and depression, based on data from the Irish Longitudinal Study on Ageing (TILDA). For grandparents caring for grandchildren pre‐pandemic in England, Di Gessa, Bordone and Arpino (2022) also found evidence that ceasing this caring role during the pandemic was associated with *poorer* mental health compared with grandparents who continued to care for their grandchildren.

A further quantitative longitudinal design used to examine caring during the COVID‐19 pandemic was to gather data at multiple time points during the period of public health restrictions. For example, Mak, Bu and Fancourt (2022) compared carers and non‐carers on multiple measures of mental health at six time points from late March/early April 2020 to the easing of most restrictions in England in late July/early August 2021. At several time points and after controlling for covariates using a propensity score‐matched analysis, carers reported more depression and anxiety compared with non‐carers but no differences for loneliness or life satisfaction.

We found few studies adopting quantitative longitudinal designs that focused on family carers of people with intellectual disabilities in relation to the COVID‐19 pandemic. Bailey, Hastings and Totsika (2021) made use of a cohort study where parents of children with intellectual disabilities provided data about their own psychological distress, life satisfaction, caring impact and positive gain pre‐pandemic. A follow‐up data collection wave was underway preceding the pandemic and continued into the period of public health restrictions. Parental outcomes were compared for participants who completed the follow‐up survey before or during the pandemic whilst controlling for earlier parent outcomes. There was no evidence of differences in parental wellbeing between the groups of parents who completed the follow‐up survey during versus prior to the COVID‐19 pandemic. Zonneveld, van Schelven and Boeije (2022) gathered quality‐of‐life and care burden data from carers of adults with intellectual disabilities in 2019 and at two points during the pandemic. Although a small number of participants (*n* < 40) completed questionnaires at all three data collection points, no repeated measures analyses were reported. The authors did, however, report that there were no changes in carer quality of life between the data collection points, but higher self‐reported ‘care burden’ during the pandemic data collection. Tarzi et al. (2023) compared wellbeing in two different groups of family carers who completed measures prior to vaccines being available and a group completing measures in April or June 2022 with the latter group (further into the lifting of restrictions) reporting higher levels of wellbeing. Across the three longitudinal studies, outcome measures varied: psychological distress, Kessler 6 (K6; Kessler et al. 2002), Warwick–Edinburgh Mental Wellbeing Scale (WEMWBS; Stewart‐Brown et al. 2009) and a Dutch version of the WHOQOL‐BREF (De Vries and Van Heck 1996; WHO 1996).

All three of these ‘longitudinal’ quantitative studies have limitations (such as only two time points available, small samples, and interrupted longitudinal design rather than a full cohort follow‐up) that make it difficult to definitively identify whether the COVID‐19 pandemic and associated restrictions had an impact on family carers of people with intellectual disabilities nor how family‐carer outcomes may have changed at different times during the pandemic. The purpose of the current study was to examine how trajectories of family‐carer wellbeing and the impact of the caring role on carers' health changed over four time points measured during the period of the COVID‐19 pandemic. We examined wellbeing and changes in impact of the caring role on carers' health trajectories of family carers across the United Kingdom between early autumn 2020 and early autumn 2022. We also explored associations between a number of key covariates (profound and multiple intellectual disabilities, the cared‐for person's individual wellbeing, the cared‐for person's age, whether the cared‐for person lived with their family) and family‐carer wellbeing and the impact of the caring role on carers' health trajectories.

## 1 Methods

### 1.1 Design

This study uses data collected from the UK longitudinal ‘Coronavirus and people with intellectual disabilities cohort study’ (Hatton et al. 2023; Flynn et al. 2021), which recruited family carers of people with intellectual disabilities and people with intellectual disabilities themselves across four waves of data collection: during the winter period of 2020/21 (mainly during lockdown restrictions; Wave 1), across the spring of 2021 (some public health protection measures were being lifted during this period; Wave 2), in late summer 2021 (protective measures had mostly been removed; Wave 3) and summer/early autumn 2022 (all protection measures had been removed; Wave 4).

### 1.2 Participants

The original study recruited two cohorts of individuals: Adults with intellectual disabilities who could respond for themselves, and a second cohort of family carers or support staff of people with intellectual disabilities who were unable to be interviewed directly themselves (see Hatton et al. 2021 for details). In the current study, we focused on 312 family carers of a person with intellectual disabilities from the second cohort. This sample consisted of 211 adults with intellectual disabilities (69%) living in the family home and the remaining 97 (31%) living in other group living/residential accommodation. The purpose of the survey was primarily to ask family carers to report about the COVID‐19 experiences of an adult with intellectual disabilities.

In addition, some data about outcomes for family carers were collected and these data are the focus of the current report. Due to the main focus of the study on the adults with intellectual disability, specific demographic information about family carers was not gathered. One hundred and seventy (55%) of the people with intellectual disabilities were male, 134 (43%) were female and 6 were reported as identifying with other genders or did not want to respond (1.9%). The average age of adults with intellectual disabilities was 30.5 years (SD = 11.7 years). Approximately, 92% of the people with intellectual disabilities had a White ethnic background, with the remainder of the sample belonging to an ethnic minority group (Black, Asian Chinese, mixed race and other). One hundred and thirty‐nine (50%) of the adults with intellectual disabilities were reported by their family carer as having ‘profound and multiple learning (intellectual) disabilities’ (PMID). Family carers were regionally spread across the United Kingdom with 33.65% from England, 13.46% from Northern Ireland, 34.3% from Scotland and 18.59% from Wales.

### 1.3 Outcome Measures

The Short Warwick‐Edinburgh Mental Wellbeing Scale (SWEMWBS; Stewart‐Brown et al. 2009) is a seven item self‐report measure of mental wellbeing; the shortened version of the 14 item WEMWBS that was developed and validated using clinical samples (Tennant et al. 2007; Shah et al. 2021). Higher scores indicate better wellbeing. The measure has good psychometric properties with weighted kappa between 0.79 and 0.85 (Ng Fat et al. 2017) and high internal consistency, Cronbach's *α* = 0.88 (Ringdal et al. 2018).

The impact of caring role on family‐carers' health composite score was assessed in the study survey, family carers were routinely asked at each data collection wave whether, ‘In the last four weeks, has your health been affected by your caring role in any of the following ways..’ with options: feeling tired, feeling depressed, loss of appetite, disturbed sleep, general feelings of stress, physical strain (e.g., back), short tempered or irritable, had to contact own GP, developed my own health conditions, or made an existing condition worse. These questions were drawn from the Personal Social Services Survey of Adult Carers in England, 2021–2022 (NHS Digital 2022). The binary indicators for each item checked were summed to form an index of care burden. At Wave 1, the Kuder Richardson coefficient (Anselmi, Colledani and Robusto 2019) was 0.82, indicating an acceptable level of reliability. A value of zero that indicated no health impacts as a result of their caring role was identified by the family carer, and higher scores were indicative of a greater level of the impact of caring role on family‐carers' health.

### 1.4 Predictor Variables

One time‐varying and four fixed predictors were used in the analysis. Fixed factors were whether the person with intellectual disabilities was described as having PMID (or not), the age of the person with intellectual disabilities in years at Wave 1, whether the person with intellectual disabilities at Wave 1 was living in the family home with the caregiver (or not), and data about the person with intellectual disabilities' perceived mental health at Wave 1 (summed score from three 3‐point rated survey questions: ‘Compared to before start of the first lockdown in March 2020, do you think the person you support/care for has been feeling more or less …’: worried or anxious, sad or down, or angry or frustrated).

The time‐varying factor at Waves 2–4 was the reported perceived mental health of the person with intellectual disabilities at the time of each survey. This was measured using the same three items as the Wave 1 measure but focused on perceived mental health in the preceding 4 weeks. Summed scores were generated for each Waves 2–4.

### 1.5 Procedure

Recruitment of participants was through multiple pathways across the United Kingdom: collaborating organisations in each of the four UK nations, social media and wider networks of intellectual disabilities and family organisations in England, Northern Ireland, Scotland and Wales. Potential participants followed a direct link to an online survey using Qualtrics on the research project website. The survey included study information and consent questions before the survey started. No participants received an honorarium for participating. Data were gathered over four waves from 2020–2022 (see Section 1.1).

### 1.6 Statistical Analysis

A multilevel model framework was used to account for the dependency of observations for individuals' repeated measures; time (wave of data collection—Level 1) was nested within individuals (Level 2). Within this framework, two models were fitted to account for different coding of the perceived mental health variable at Wave 1 compared with other waves. The first model included the perceived mental health of the person with intellectual disabilities measured at Wave 1 as a time‐invariant predictor and used all waves of data in the outcome measures (Waves 1–4) whereas the second model included the perceived mental health of the person with intellectual disabilities as a time‐varying predictor between Waves 2 and 4 and outcomes measured at the corresponding waves. Additionally, the following covariates relating to the person with intellectual disabilities about whom family carers were reporting were included at Level 2 (individual): PMID, age of person with intellectual disabilities and whether they lived in the family home with the family carer.

A linear mixed effects model was fitted to the WEMWBS total score outcome, but a generalised linear mixed model with zero inflation was used for the impact of caring role on family‐carers' health outcome, given the skew in the outcome measure and the higher number of zero values. Models allowed for missingness at one time point to remain in the model and used maximum likelihood estimates. There was a considerable amount of missingness in the WEMWBS total score with around 44% missing, but only 1% missing for the impact of caring role on family‐carers' health scores. Predictor variables contained varying amounts of missingness, up to 21%, so multiple imputation was used to perform a sensitivity analysis for the primary analysis conducted. Data were imputed for the Level 2 variables using full conditional specification and specifically fitted models using either predictive mean matching, Poisson, binary or normal distribution options. Five imputed datasets were created and up to 10 iterations used to ensure convergence.

All analyses used R statistical software (R Core Team 2024) with the R packages ‘lme4’,‘lmerTest’, ‘glmmTMB’ and ‘Performance’. Multiple imputation was conducted using the R packages ‘mice’, ‘miceadds’ and ‘countimp’.

### 1.7 Ethical Approval

The Manchester Metropolitan University Faculty of Health, Psychology and Social Care Faculty Research Ethics Committee provided research ethical review and approval for this study.

## 2 Results

Means and standard deviations for family‐carer outcomes and time‐varying perceived mental health of the person with intellectual disabilities are presented in Table 1. The family‐carer wellbeing measure, WEMWBS, was mostly static across the first three waves with a notable uptick in wellbeing at Wave 4. The impact of caring role on family‐carers' health composite measure showed a gradual decline across the four time points (mean difference = 1.27 points or Cohens' *d* = 0.51 between Waves 1 and 4), potentially reflecting an easing in carer responsibilities and associated improvement in family carers' health as pandemic measures were lifted. Within‐person mental health scores for the person with intellectual disabilities declined across time reflecting improved mental health for this sub‐sample of the total study from Waves 2 to 4.

**TABLE 1:** Means and standard deviations for mental health predictors and family‐carer outcomes at each wave of measurement.

	Wave 1	Wave 2	Wave 3	Wave 4
Variable	Mean (SD)	Mean (SD)	Mean (SD)	Mean (SD)
Outcome				
WEMWBS	20.0 (3.40)	20.6 (3.79)	20.3 (3.41)	22.3 (4.37)
Impact of caring role on family‐carers' health	2.72 (2.63)	1.85 (2.43)	2.04 (2.47)	1.45 (2.36)
Within‐person predictor				
Mental health of person with intellectual disabilities	—	5.85 (1.66)	5.82 (1.69)	5.70 (1.58)
Between‐person predictor				
Mental health of person with intellectual disabilities	4.08 (1.26)	—	—	—

### 2.1 Family‐Carer Wellbeing: Using All Waves of Data but Considering the Person With Intellectual Disabilities Wellbeing at Wave 1 Only (Model 1)

We found that there was a small increase in family‐carer wellbeing across time ( β = 0.58, 95% CI [0.43, 0.73], *p* < 0.001; see Table 2). There was a non‐significant reduction in wellbeing for family carers of people with PMID compared with those without PMID ( β = − 0.09, 95% CI [−0.90, 0.72], *p* = 0.819). Better reported mental health of the person with intellectual disabilities at Wave 1 was associated with increased carer wellbeing ( β = 0.74, 95% CI [0.42, 1.06], *p* < 0.001). There was no reliable association between family‐carer wellbeing and whether the person with intellectual disabilities lived in the family home.

**TABLE 2:** Parameter estimates for family‐carer wellbeing growth model using waves 1–4 data.

Predictors (all re the person with intellectual disabilities)	WEMWBS (model 1)			WEMWBS (model 2)		
	Estimates	CI	*p*	Estimates	CI	*p*
(Intercept)	15.34	[13.26, 17.42]	**< 0.001**	22.22	[19.95, 24.50]	**< 0.001**
Time	0.58	[0.43–0.73]	**< 0.001**	0.79	[0.52, 1.06]	**< 0.001**
PMID	−0.09	[−0.90, 0.72]	0.819	0.22	[−0.72, 1.15]	0.649
Wave 1 mental health	0.74	[0.42, 1.06]	**< 0.001**	—	—	—
Age (years)	0.03	[−0.01, 0.07]	0.123	0.04	[0.00, 0.08]	0.071
Lives with family	0.05	[−0.85, 0.95]	0.912	−0.16	[−1.20, 0.89]	0.768
Mental health (time varying)	—	—	—	−0.55	[−0.77, −0.34]	**< 0.001**
Random effects						
σ^2^	4.09			4.45		
τ_00_	7.95_PID_			9.00_PID_		
ICC	0.66			0.67		
*N*	232_PID_			204_PID_		
Observations	662			444		
Marginal *R* ^2^/conditional *R* ^2^	0.103/0.695			0.099/0.702		

*Note:* Wave 1 individual mental health of the person with intellectual disabilities included as a time‐invariant predictor (model 1). Individual mental health of the person with intellectual disabilities included as a time‐varying predictor between Waves 2 and 4 (Model 2).

### 2.2 Family‐Carer Wellbeing: Using Waves 2–4 Data and Considering Individual Mental Health of the Person With Intellectual Disabilities as Time Varying (Model 2)

We found that there was a small increase in family‐carer wellbeing across time ( β = 0.79, 95% CI [0.52, 1.06], *p* < 0.001; see Table 2). The cared‐for person with intellectual disabilities' mental health included as a time‐varying predictor was also found to be predictive of family‐carer wellbeing. Family carers supporting a person with intellectual disabilities and more positive mental health had increased wellbeing ( β = − 0.55, 95% CI [*−*0.77, −0.34], *p* < 0.001). We found that there was no evidence of an association between any other predictors and carer wellbeing.

### 2.3 Impact of Caring Role on Family‐Carers' Health: Using All Waves of Data but Considering the Person With Intellectual Disabilities' Mental Health at Wave 1 Only (Model 3)

There was a small decrease in the impact of caring role on family‐carers' health across time, around a 6% reduction between waves (IRR = 0.95, 95% CI [0.91, 0.99], *p* = 0.009; see Table 3). The impact of caring role on family‐carers' health increased by around 1.24 times for family carers of people with PMID compared with those without PMID (I*RR* = 1.24, 95% CI [1.07, 1.45], *p* = 0.015). Better reported mental health of the person with intellectual disabilities at Wave 1 was associated with reduced impact of caring role on family‐carers' health (IRR = 0.87, 95% CI [0.82, 0.93], *p* < 0.001). When the person with intellectual disabilities lived in the family home, the impact of caring role on family‐carers' health was also higher (IRR = 1.38, 95% CI [1.15, 1.65], *p* < 0.001). The age of the person with intellectual disabilities was not a significant correlate of the impact of caring role on family‐carers' health.

**TABLE 3:** Parameter estimates for the impact of caring role on family‐carers' health growth model using Waves 1–4 data.

Predictors (all re the person with intellectual disabilities)	Impact of caring role on family‐carers' health (Model 3)			Impact of caring role on family‐carers' health (Model 4)		
	Incidence rate ratios	CI	*p*	Incidence rate ratios	CI	*p*
(Intercept)	5.81	[3.89, 8.68]	**< 0.001**	0.94	[0.60, 1.49]	0.793
Time	0.95	[0.91, 0.99]	**0.009**	1.07	[1.00, 1.14]	0.055
PMID	1.24	[1.07, 1.45]	**0.005**	1.35	[1.15, 1.60]	**<0.001**
Wave 1 mental health	0.87	[0.82, 0.93]	**< 0.001**	—	—	—
Age	0.99	[0.99, 1.00]	0.8	0.99	[0.99, 1.00]	0.124
Lives with family	1.38	[1.15, 1.65]	**0.001**	1.55	[1.27, 1.88]	**< 0.001**
Mental health (time varying)	—	—	—	1.16	[1.11, 1.21]	**< 0.001**
Zero‐inflated model						
(Intercept)	0.49	[0.41, 0.58]	**< 0.001**	0.02	[0.01, 0.06]	**< 0.001**
Random effects						
σ^2^	0.38			0.91		
τ_00_	0.18 _PID_			0.19 _PID_		
ICC	0.32			0.18		
*N*	238 _PID_			206 _PID_		
Observations	947			457		
Marginal *R* ^2^/conditional *R* ^2^	0.129/0.408			0.112/0.269		

### 2.4 Impact of Caring Role on Family‐Carers' Health: Using Waves 2–4 Data and Considering the Person With Intellectual Disabilities' Mental Health as Time Varying (Model 4)

We found that there was no statistically significant change in the impact of caring role on family‐carers' health across time (RR = 1.07, 95% CI [1.00, 1.14], *p* = 0.055; see Table 3). As previously found, the impact of caring role on family‐carers' health increased by around 1.35 times for family carers of people with PMID compared with those without PMID (RR = 1.35, 95% CI [1.15, 1.60], *p* < 0.001). Better reported mental health of the person with intellectual disabilities across time was associated with reduced impact of the caring role on family‐carers' health (RR = 1.16, 95% CI [1.11, 1.21], *p* < 0.001). When the person with intellectual disabilities lived in the family home, impact of the caring role on family‐carers' health was also higher (RR = 1.55, 95% CI [1.27, 1.88], *p* < 0.001).

### 2.5 Missing Data Sensitivity Analyses

After accounting for missing data via a sensitivity analyses, we found that the pattern of results and magnitudes of estimates were almost identical in all models (see Tables S1, S3 and S4). In Model 2, we found that the pattern of results was similar, with the exceptions of the time and mental health parameters reducing in magnitude and becoming non‐significant ( β = 0.27, 95% CI [−0.47, 1.00], *p* = 0.481; and β = − 0.45, 95% CI [−0.90, −0.01], *p* = 0.055, respectively; see Table S2).

## 3 Discussion

We explored the trajectories of wellbeing and the impact of the caring role on family‐carers of people with intellectual disabilities' health, using two implementations of the statistical models (either with mental health of the person with intellectual disabilities as a time‐varying or time‐invariant predictor). In each implementation of the models for each outcome (and including sensitivity analyses for missing data), we found small increases in family‐carer wellbeing and reductions in the impact of the caring role on family‐carers' health across the COVID‐19 pandemic time course (i.e., the four waves of the study). There are no directly comparable data for family carers of adults with intellectual disabilities, but these findings are in concert with those of Tarzi et al. (2023) whose data suggested better outcomes for family carers at a later point during the pandemic period (presumably when more restrictions were lifted). Due to the initial data collection point in our study being during the first extended period of restrictions and subsequent time points collected after public health measures were being relaxed, the positive changes noted may relate to improvements in the limitation on access to services and the reduction of social restrictions.

The additional unique element of the current study was the opportunity to examine some factors that may be associated with carer wellbeing and impact of the caring role on family‐carers' health over the course of the pandemic period. Both statistical models for the impact of the caring role on family‐carers' health showed that caring for someone with PMID was associated with increased impact of the caring role on family‐carers' health but did not reduce family‐carer wellbeing. These findings are consistent with existing data indicating that the care demands associated with PMID might lead to additional stress for family carers (e.g., Linden et al. 2022). In addition, there were additional care challenges during the COVID‐19 pandemic for families supporting people with PMID including limited access to healthcare and the need to further protect those with vulnerable health (i.e., ‘shielding’ in the United Kingdom) (Bradshaw et al. 2024). Furthermore, families of adults with PMID had limited access to staff who may normally have assisted with caregiving as well as additional complexity around managing illness.

We also expected, based on family systems theory assumptions and existing research findings (Hastings 2016), that there would be associations between the wellbeing of the person with intellectual disabilities and family‐carer wellbeing and impact of the caring role on family‐carers' health during the COVID‐19 pandemic period. The initial reported impact of reduced mental health of the cared‐for person (as reported retrospectively at Wave 1) and time‐varying mental health of the cared‐for person (Waves 2–4) were both associated with reduced carer wellbeing and increased impact of the caring role on family‐carers' health. It is important to note that the association with time‐varying mental health of the cared‐for person was not found once we had accounted for the effects of missing data. As might be expected, when the cared‐for person lived in the family home, family carers reported greater impact of the caring role on family‐carers' health—presumably because more day‐to‐day care was being carried out by the family carer in these circumstances. Interestingly, family‐carer wellbeing was not related to whether the cared‐for person was living in the family home.

### 3.1 Limitations

Although this study has several benefits including relatively large sample size for this population and recruiting during a pandemic, there are some potential limitations to findings. Given that the cared‐for person's wellbeing questions at Wave 1 were changed in subsequent waves, we fitted models with and without the cared‐for person's wellbeing as a time‐varying predictor to reflect the data structures. Retrospectively, this could have been kept consistent, and then time varying only predictor models could have been fitted. A further limitation is that due to a convenience study sampling design, pre‐pandemic data could not be collected, which would have been more informative to address questions on change from pre‐pandemic levels. Similarly, given that this is a convenience sample, our ethnicity breakdown (for the person with intellectual disabilities) is mainly a White sample, limiting our ability to explore intersection between ethnicity and caregiving responsibility. It is also likely that certain groups of carers less able to navigate the online environment or those struggling the most with their own health will have been excluded from the study and so findings should be interpreted with that in mind. Lastly, given that the focus of the main study was data collection from people with intellectual disabilities themselves rather than their carers, there was limited information on carers' demographics.

### 3.2 Implications

Although the study from which the data were drawn was not designed to examine whether the COVID‐19 pandemic and associated public health restrictions had an (perhaps assumed negative) impact on family carers of adults with intellectual disabilities, the findings do have some implications for support of family carers. First, family carers of people with PMID may face significant additional care demands and services and policy makers would need to carefully consider the support needs of these families in future pandemics and outside of the pandemic context. Second, the mental health of the cared‐for person with intellectual disabilities was a key predictor of family‐carer wellbeing and impact of the caring role on family‐carers' health. Thus, additional focus on supporting the mental health of adults with intellectual disabilities in future pandemics—perhaps especially those with more severe to profound intellectual disabilities as was thought to have suffered due to a general lack of stimulation and support, arising from the COVID restrictions and a loss of support (likely the main sample for the present analysis)—would be doubly important as such efforts may also contribute to family carers' own wellbeing.

Finally, although we found increases in family‐carer wellbeing and reduced impact of the caring role on family‐carers' health over the four study waves (presumably associated with reducing public health restrictions over time) these changes were small. Thus, more research is required to understand the initial impact of social and public health restrictions and changes over time as public health restrictions change. Given we had no pre‐pandemic measurement point, it is not clear if the small improvements observed returned family carers to pre‐pandemic levels of wellbeing and impact of the caring role on family‐carers' health or if wellbeing by Wave 4 was still lower and impact of the caring role on family‐carers' health higher than was typical for this group. The current data do suggest that more could and should be done to offer direct support to help improve family‐carer wellbeing and impact of the caring role on family‐carers' health during and coming out of any future pandemics.

## Ethics Statement

The Manchester Metropolitan University Faculty of Health, Psychology and Social Care Faculty Research Ethics Committee provided research ethical review and approval for this study.

## Conflicts of Interest

The authors declare no conflicts of interest.

## Supporting information


**Table S1** Sensitivity analysis for missing data using multiple imputation for model 1 carer wellbeing (WEMWBS).
**Table S2** Sensitivity analysis for missing data using multiple imputation for model 2 carer wellbeing (WEMWBS).
**Table S3** Sensitivity analysis for missing data using multiple imputation for model 3 Impact of caring role on family‐carers ’ health.
**Table S4** Sensitivity analysis for missing data using multiple imputation for model 4 Impact of caring role on family‐carers ’ health.

## Data Availability

A quantitative dataset will be archived online in a form that will be available to researchers after all waves of data collection for the project have been completed.
