# Supplementary material for: Wellbeing of Family Carers of Adults With Intellectual Disabilities During the COVID‐19 Pandemic in the UK: Longitudinal Study
Source: J Intellect Disabil Res. 2024 Dec 24;69(4):265–73. doi: 10.1111/jir.13206 (PMC11876487; doi:10.1111/jir.13206)
Supplement: Supplementary file 1 — Table S1 Sensitivity analysis for missing data using multiple imputation for model 1 carer wellbeing (WEMWBS). Table S2 Sensitivity analysis for missing data using multiple imputation for model 2 carer wellbeing (WEMWBS). Table S3 Sensitivity analysis for missing data using multiple imputation for model 3 Impact of caring role on family‐carers ’ health. Table S4 Sensitivity analysis for missing data using multiple imputation for model 4 Impact of caring role on family‐carers ’ health. [file JIR-69-265-s001.docx]

**Supplementary file**

**Supplementary Table S1** Sensitivity analysis for missing data using multiple imputation for model 1 carer wellbeing (WEMWBS).

|  | **WEMWBS (model 1)** | | |
| --- | --- | --- | --- |
| *Predictors* | *Estimates* | *CI* | *P* |
| (Intercept) | 17.25 | 15.61 – 18.89 | **<.001** |
| time | 0.24 | 0.11 – 0.36 | **<.001** |
| PMLD | -0.14 | -0.78 – 0.50 | **.666** |
| W1 mental health | 0.59 | 0.35 – 0.83 | **<.001** |
| Age | 0.02 | -0.01 – 0.05 | .197 |
| Lives with family | -0.23 | -0.98 – 0.53 | .561 |

**Supplementary Table S2** Sensitivity analysis for missing data using multiple imputation for model 2 carer wellbeing (WEMWBS).

|  | **WEMWBS (model 2)** | | |
| --- | --- | --- | --- |
| *Predictors* | *Estimates* | *CI* | *P* |
| (Intercept) | 22.55 | 18.87 – 26.23 | **<.001** |
| time | 0.27 | -0.47 – 1.00 | .481 |
| PMLD | 0.16 | -0.90 – 1.23 | .765 |
| mental health (TVC) | -0.45 | -0.90 – -0.01 | .055 |
| Age | 0.02 | -0.03 – 0.07 | .358 |
| Lives with family | -0.19 | -1.36 – 0.98 | .753 |

**Supplementary Table S3** Sensitivity analysis for missing data using multiple imputation for model 3 Impact of caring role on family-carers’ health.

|  | **Impact of caring role on family-carers’ health (model 3)** | | |
| --- | --- | --- | --- |
| *Predictors* | *Estimates* | *CI* | *P* |
| (Intercept) | 5.04 | 3.50 – 7.26 | **<.001** |
| time | 0.96 | 0.92 – 0.99 | **.023** |
| PMLD | 1.22 | 1.06 – 1.40 | **.006** |
| W1 mental health | 0.90 | 0.84 – 0.95 | **<.001** |
| Age | 1.00 | 0.99 – 1.00 | .205 |
| Lives with family | 1.32 | 1.12 – 1.56 | **<.001** |
| **Zero-Inflated Model** |  |  |  |
| (Intercept) | 0.65 | 0.57 – 0.75 | **<.001** |

**Supplementary Table S4** Sensitivity analysis for missing data using multiple imputation for model 4 Impact of caring role on family-carers’ health.

|  | **Impact of caring role on family-carers’ health (model 4)** | | |
| --- | --- | --- | --- |
| *Predictors* | *Estimates* | *CI* | *P* |
| (Intercept) | 1.05 | 0.56 – 1.96 | .886 |
| time | 1.03 | 0.97 – 1.10 | .366 |
| PMLD | 1.26 | 1.08 – 1.48 | **.004** |
| mental health (TVC) | 1.16 | 1.08 – 1.25 | **.002** |
| Age | 1.00 | 0.99 – 1.00 | .294 |
| Lives with family | 1.41 | 1.16 – 1.72 | **<.001** |
| **Zero-Inflated Model** |  |  |  |
| (Intercept) | 0.81 | 0.65 – 1.00 | .006 |
